# Combination of Alprazolam and Bailemian Capsule Improves the Sleep Quality in Patients With Post-Stroke Insomnia: A Retrospective Study

**DOI:** 10.3389/fpsyt.2019.00411

**Published:** 2019-06-07

**Authors:** Jian Wang, Zhiqiang Wang, Xiaoyan Wang, Guo Du, Bo Zheng, Yuxia Li, Qingsong Wang

**Affiliations:** ^1^Department of Neurology, Yaan People’s Hospital, Yaan, China; ^2^Department of Neurology, The General Hospital of Western Theater Command, Chengdu, China

**Keywords:** stroke, insomnia, drug therapy, Bailemian capsule, alprazolam

## Abstract

Insomnia is often ignored in the diagnosis and treatment of patients of stroke. The present study aimed to evaluate the efficacy of alprazolam (ALP) combined with Bailemian capsule (BC, a traditional Chinese patent medicine) in the treatment of post-stroke insomnia (PSI). A total of 231 stroke patients involved in this retrospective study were treated with ALP, BC, or ALP + BC for 3 weeks. The quality of sleep was evaluated by the Pittsburgh Sleep Quality Index (PSQI) and polysomnography (PSG), while self-care ability was monitored by the modified Rankin Scale (mRS) before and after treatment. Compared with the baseline, the self-care ability of patients in each group was significantly improved after treatment (*P* < 0.01). The PSQI data showed a significant improvement in all patients in all of the subjective PSQI items and global score (*P* < 0.05). Notably, ALP + BC administration had a significantly greater effect on sleep latency, quality, disturbance, and efficiency, as well as daytime dysfunction and global PSQI than the use of ALP or BC alone (*P* < 0.05). The PSG data showed that ALP significantly improved the sleep efficiency and decreased the arousal times, rapid eye movement (REM) sleep, and sleep latency (*P* < 0.05), while BC significantly improved the sleep efficiency, total sleep time, and the duration of N3 (*P* < 0.05). Strikingly, ALP + BC achieved the effect of both ALP and BC (*P* < 0.05). Importantly, the effect of the combination of ALP and BC was greater than the use of ALP or BC alone, which was consistent with the result of PSQI. In conclusion, the sleep quality and self-care ability of patients with PSI were improved by ALP and BC, thereby supporting the potential advantages of ALP combined with BC in the treatment of patients with PSI.

## Introduction

Stroke is the second most common cause of deaths worldwide (1) as well as the leading cause of long-term disability (2, 3). Post-stroke insomnia (PSI) is a common symptom but often underestimated and is even ignored in the diagnosis and treatment (4). Up to 70% of the patients with acute stroke have sleep disorders including excessive daytime sleepiness, insomnia, hypersomnia, and fatigue (5). PSI affects the functional recovery of the nervous system, aggravates the existing diseases such as hypertension and diabetes, and deteriorates the quality of life (6). Accumulating evidence demonstrated that poor quality of sleep could be detrimental to the immune system (7), delay the recovery (8), increase pain sensitivity (9), lead to depression and anxiety (10), and affect the functional well-being (11). Although the consequences of PSI and the potential clinical impact are severe, the condition is not well treated.

Reportedly, alprazolam (ALP) was the most commonly used Western drug in China due to its effectiveness on generalized anxiety, panic attacks with or without agoraphobia, and depression (12). ALP is a derivative of benzodiazepine, and the mechanism underlying the activity of the drug and the side effects have been described previously (13). Notably, ALP presents excessive side effects when more than 0.5 mg was used each time (14).

Drugs for stroke from traditional Chinese medicine have been developed (15, 16). According to clinical and basic research in traditional Chinese medicine, these drugs were beneficial in the prevention and treatment of stroke. BC is a traditional Chinese patent medicine. It can improve sleep quality and alleviate insomnia by elevating the level of brain contents 5-HT and GABA (17, 18). Previous studies have shown that sleep disorders were closely related to the decreased content of 5-HT and gamma-aminobutyric acid (GABA) in the central nervous system (19). The data were monitored, which showed that this drug might cause nausea, abdominal pain, rash, and itching. Despite mild adverse reactions, BC can alleviate PSI and anxiety, resulting in the improvement of life quality (20).

In this retrospective study, we evaluated the effect of BC, ALP, and BC combined with ALP on sleep quality and stroke outcome (self-care ability) in PSI patients.

## Patients and Methods

### Participants

In the present retrospective study, we analyzed 231 patients (78 females and 153 males) who were hospitalized for stroke at the Department of Neurology in the General Hospital of Western Theater Command from January 1, 2014, to September 1, 2015. All the patients underwent identical treatment for stroke according to the guidelines of acute ischemic stroke (21). The degrees of insomnia and neurological impairment were evaluated after admission. This study has been approved by the Ethics Committee of The General Hospital of Western Theater Command. Also, we obtained the informed consent of patients and their families.

The patient selection process was applied according to the following criteria:

*Inclusion criteria:* 1) The diagnosis of stroke is based on clinical presentation, computerized tomography, and/or magnetic resonance imaging scan of the brain when the stroke occurred within 7 days prior to the admission. 2) Patients had varying degrees of insomnia [*Diagnostic and Statistical Manual of Mental Disorders, Fourth Edition* (*DSM-IV*) criteria and the three insomnia-related items of the Hamilton depression scale] and received ALP, BC, or both during hospitalization. 3) Insomnia was present after stroke. 4) The score was at least 26 on the Mini-Mental State Exam. 5) The questionnaire was completed independently. And 6) antipsychotic medications, if administered, had been discontinued for more than 7 days.

*Exclusion criteria:* 1) Patients had a history of sleep disorders (based on the sleep disorders questionnaire designed in Hong Kong) (22). 2) Patients also presented with cardiovascular, liver, kidney, or any severe life-threatening diseases. 3) Patients had active epilepsy and unable to complete the entire treatment process. 4) Patients had language barrier and could not cooperate with the researchers. 5) Patients had typical mental diseases, such as anxiety and depression. 6) Patients had family history of mental disorders. 7) Patients had other severe diseases and could not complete the treatment and investigation. 8) Patients had restless legs syndrome, obstructive sleep apnea–hypopnea syndrome, central sleep apnea syndromes, and rapid eye movement (REM) sleep behavior disorder.

## Study Design

The patients were divided into three groups according to the treatment. The patients received ALP (12) (*n* = 71, 0.4 mg/day, taken 30 min before sleep at night; Qilu Pharmaceutical Group, Jinan, Shandong, China, national drug approval number: H37021277), BC (20) (*n* = 87, four capsules each time after breakfast and dinner; Yangtze River Pharmaceutical Co., Ltd, Taizhou, Jiangsu, China, national drug approval number: Z20020131), or ALP + BC (*n* = 73) for 3 weeks. Pittsburgh Sleep Quality Index (PSQI) and modified Rankin Scale (mRS) were applied to evaluate the sleep quality and the self-care ability of patients before and after treatment. Furthermore, we also objectively assessed the data of polysomnography (PSG) before and after treatment in order to evaluate the curative effect impersonally (*n* = 5 in each group).

## Assessment Criteria and Questionnaires

*Sleep quality:* PSQI is a self-reported questionnaire tool for subjectively measuring the quality of sleep for adults in the last month. It includes seven sleep-related items, such as latency, quality, duration, disturbances, efficiency, the use of sleep medications, and daytime dysfunction. Each item is assigned a score of 0–3 points so that the total score of PSQI is 0–21. A total score of 7 means good sleep, 7–11 points indicate mild, 12–16 points moderate, and 17–21 points severe sleep disorder (23).

*PSG:* PSG was performed as reported previously (24). Two nights of PSG recording were recommended; however, the first night was regarded as the “adaptation” night and removed from the analysis as it was not representative of the usual sleeping patterns (25). Briefly, PSG was performed with an eight-channel Grass electroencephalograph in one night. The recordings included brain electrical activity, eye movements, chin muscle activity, nasal and oral airflow, thoracic and abdominal respiratory movements, heart rate, and leg movements. Also, the following parameters were recorded: total recording time, total sleeping time, sleep efficiency, sleep latency, paradoxical latency, duration of non-rapid eye movement sleep (NREM) and its three stages (N1, N2, and N3), duration of rapid eye movement sleep (REM), wake time after sleep onset, and arousal time (26).

*Clinical outcomes:* The prognosis of stroke was assessed using mRS, a commonly used scale for measuring the self-care ability.

*Adverse reactions:* During the treatment, adverse effects such as headache, aggravated insomnia, blood pressure increase, hyperethism, nausea and vomiting, dizziness, palpitation, frequent urination, somnolence, and numbness were evaluated. A total of four cases presented nausea and upper abdominal discomfort in the BC group, while no adverse reactions were detected in the other two groups. Moreover, the symptoms were relieved spontaneously without any additional treatment.

## Statistical Analysis

Data analysis was carried out using SPSS 21.0 software (IBM Corp, Armonk, NY, USA). Measurement data were expressed as means ± standard deviations (SDs). One-way ANOVA, Student’s *t*-test, χ^2^ test, or Mann–Whitney *U* test was utilized for group comparison. When ANOVA revealed significant differences, least significant difference (LSD) or Dunnett’s T3 *post hoc* tests were used to identify significant differences among three groups. *P* < 0.05 was considered as a statistical significance.

## Results

### Comparison of Demographic and Clinical Characteristics Between the Three Groups

No significant difference was observed in the baseline characteristics of age, sex, blood glucose, triglyceride (TG), cholesterol (TC), High-density lipoprotein cholesterol (HDL-C), Low-density lipoprotein cholesterol (LDL-C), serum uric acid, and homocysteine between the three groups (*P* > 0.05, **Table 1**).

**Table 1:** Demographic and clinical characteristics of the patients. (Data are expressed as *n* (%) or mean ± SD.)

		ALP + BC	ALP	BC	F	P
Sex	Male	48 (65.75)	49 (69.01)	56 (32.15)	0.174	0.677
	Female	25 (34.25)	22 (30.99)	31 (41.65)		
Age, y		58 ± 10.05	56.13 ± 14.28	57.95 ± 11.98	0.565	0.569
Serum glucose, mmol/L		5.8 ± 2.11	5.47 ± 1.28	5.48 ± 1.66	0.909	0.404
TG, mmol/L		1.72 ± 0.98	1.93 ± 1.41	1.86 ± 1.57	0.467	0.627
TC, mmol/L		4.53 ± 1.23	4.42 ± 1.25	4.58 ± 1.43	0.320	0.726
HDL-C, mmol/L		1.1 ± 0.22	1.14 ± 0.22	1.47 ± 2.16	1.849	0.160
LDL-C, mmol/L		2.84 ± 0.97	2.89 ± 0.88	2.75 ± 0.92	0.450	0.638
Serum uric acid, µmol/L		305.17 ± 86.15	315.3 ± 80.42	306.98 ± 86.05	0.298	0.742
Homocysteine, µmol/L		15.03 ± 6.7	14.24 ± 5.99	13.84 ± 6.18	0.720	0.488

ALP, alprazolam; BC, Bailemian capsule; TG, triglyceride; TC, cholesterol; HDL-C; LDL-C.

### Comparison of Sleep Quality by PSQI Before and After ALP, BC, and ALP + BC Treatment

**Figure 1:**
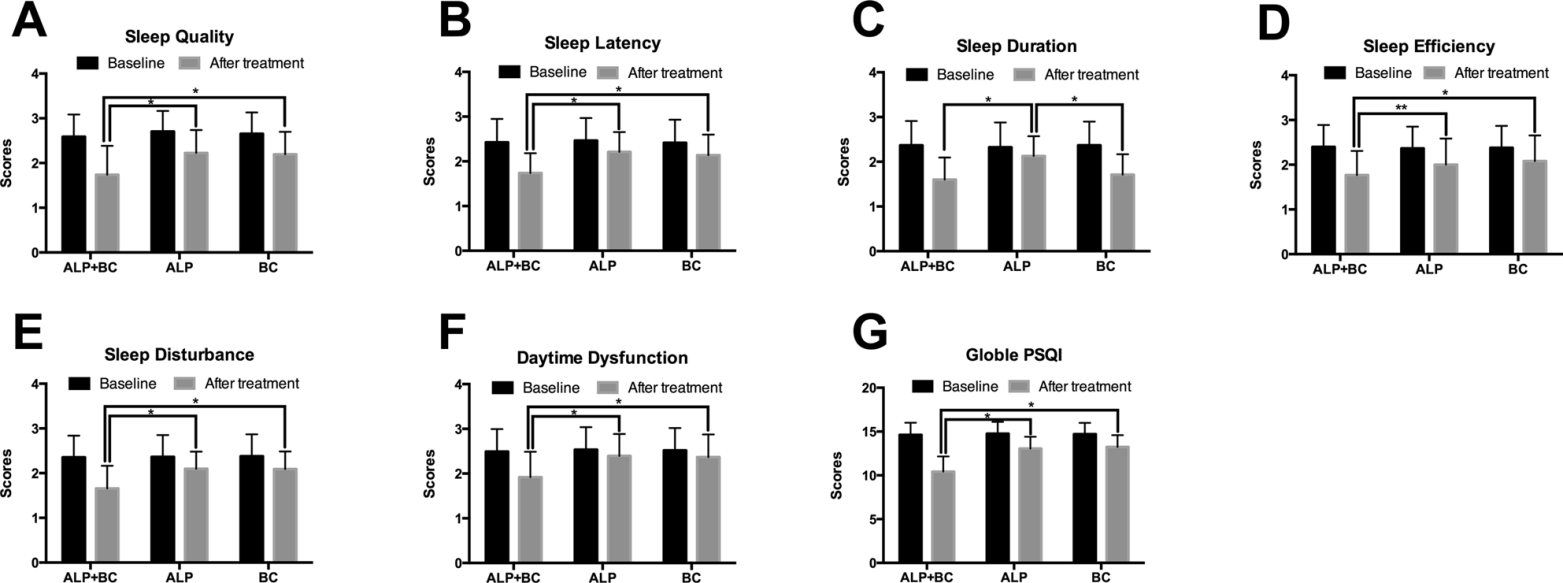
Effects of alprazolam and Bailemian on PSQI components. **(A)** Sleep quality, **(B)** sleep latency, **(C)** sleep duration, **(D)** sleep efficiency, **(E)** sleep disturbances, **(F)** daytime dysfunction, and **(G)** global score were measured according to the PSQI. *P < 0.01, **P < 0.05. PSQI, Pittsburgh Sleep Quality Index; ALP, alprazolam; BC, Bailemian capsule.

The data did not show any significant difference in the sleep quality (*F*_(2,228)_ = 1.055, *P* = 0.35), sleep latency (*F*_(2,228)_ = 0.205, *P* = 0.815), sleep duration (*F*_(2,228)_ = 0.169, *P* = 0.845), sleep efficiency (*F*_(2,228)_ = 0.074, *P* = 0.929), sleep disturbances (*F*_(2,228)_ = 0.046, *P* = 0.955), daytime dysfunction (*F*_(2,228)_ = 0.127, *P* = 0.881), sleep medication (*F*_(2,228)_ = 0.197, *P* = 0.822), and global score (*F*_(2,228)_ = 0.143, *P* = 0.867) (**Figure 1**) at baseline. After 3 weeks of treatment, all the three treatment groups significantly improved in all of the subjective PSQI items and global score (**Figure 1**, *P* < 0.05). Notably, ALP + BC administration exerted a significantly greater effect on all the PSQI items and the global PSQI score than did the other groups, except for the sleep duration, which was greatly affected by ALP + BC or BC as compared with ALP (**Figure 1C**, *P* < 0.05).

### Comparison of Sleep Quality by PSG Before and After ALP, BC, and ALP + BC Treatment

**Figure 2:**
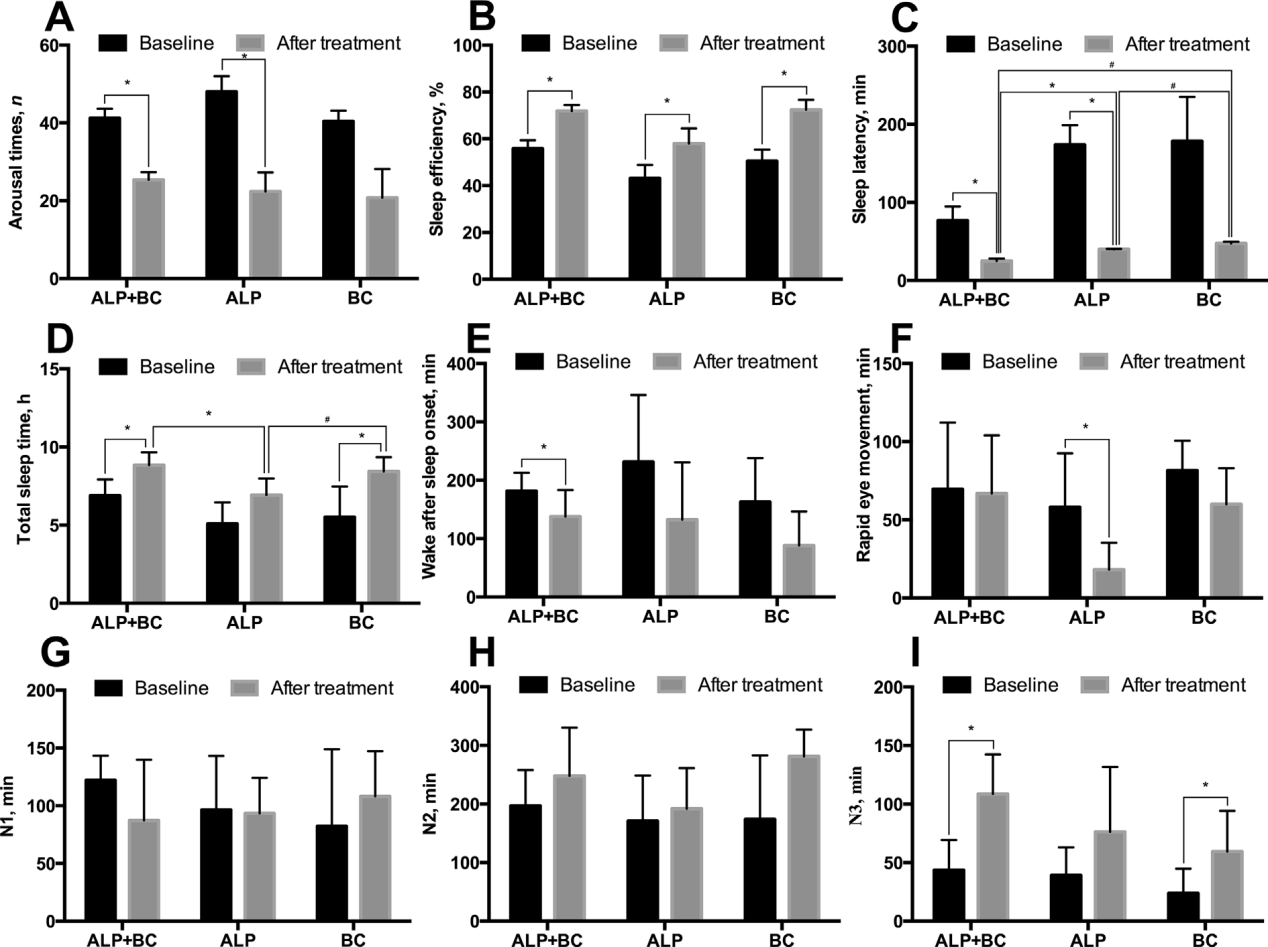
Effects of alprazolam and Bailemian on the quality of sleep in patients tested by polysomnography (PSG). **(A)** Arousal times (total number across the night), **(B)** sleep efficiency, **(C)** sleep latency, **(D)** total sleep time, **(E)** wake after sleep onset, **(F)** duration of rapid eye movement, and duration of non-rapid eye movement sleep and its three stages **(G)** N1, **(H)** N2, and **(I)** N3 were measured and compared. *P < 0.05, ^#^ *P* < 0.01. ALP, alprazolam; BC, Bailemian capsule.

Five patients in each group were selected for PSG testing. The data did not reveal any significant difference in the arousal time, sleep efficiency, sleep latency, total sleep time, wake after sleep onset, REM sleep, and NREM sleep at baseline (*P* > 0.05). After 3 weeks of treatment, ALP improved the sleep efficiency and decreased the arousal times, sleep latency, and REM sleep (**Figure 2A, B, C & F**), while BC significantly improved the sleep efficiency, total sleep time, and the duration of N3 (**Figure 2B, D & I**); ALP + BC achieved the effect of both ALP and BC (**Figure 2A, B, C, D, E & I**; *P* < 0.05). There was a significant difference in sleep latency (*F*_(2,12)_ = 28.407, *P* < 0.001) and total sleep time (*F*_(2,12)_ = 5.701, *P* = 0.018) among the three groups after treatment. *Post hoc* analysis indicated that ALP significantly decreased sleep latency compared to BC (**Figure 2C**, *P* = 0.028), while BC significantly increased total sleep time compared to ALP (**Figure 2D**, *P* = 0.026). Importantly, the effect of the combination of ALP and BC was greater than that of ALP or BC alone, which was consistent with the result of PSQI.

### Comparison of Self-Care Ability Before and After ALP, BC, and ALP + BC Treatment

**Figure 3:**
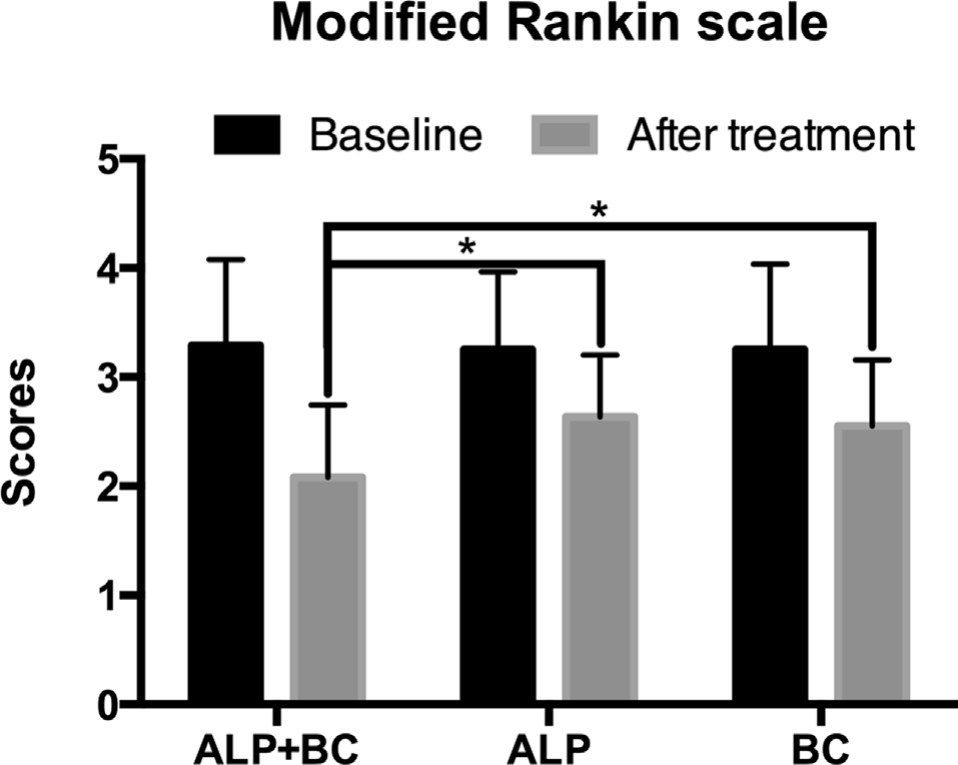
Effects of alprazolam and Bailemian on stroke outcome assessed by the modified Rankin Scale (mRS). *P < 0.001.

Previous studies showed that PSI could affect the recovery of neurological function in stroke patients (6). Therefore, we explored the effect of insomnia improvement on stroke outcome. After treatment, the stroke outcome was improved in all of the three groups, in which the mRS score decreased (*P* < 0.05). The *post hoc* study demonstrated that this effect of ALP + BC was greater than that of ALP or BC alone (**Figure 3**, *P* < 0.05).

## Discussion

The present study examined the therapeutic effect of ALP, BC, and the combined effect of ALP and BC on improving the sleep quality and self-care ability in patients with PSI. The results revealed that the oral administration of ALP and BC alone significantly but differentially improves the subjective perception of sleep quality by PSQI and objective sleep quality by PSG. However, the combined effect of ALP and BC is better than that of ALP or BC alone.

Next, compared with the baseline, ALP + BC administration had a significantly greater effect on sleep latency, quality, disturbance, and efficiency than did either ALP or BC alone, which was consistent with objective PSG and subjective PSQI. In addition, in comparison with the baseline after the use of BC or ALP, PSQI data showed that all items of PSQI were improved. Surprisingly, after 3 weeks of treatment, among the 15 patients (*n* = 5 in each group), the PSG data showed that ALP improved the sleep efficiency and decreased the arousal times, sleep latency, and REM sleep, while BC improved the sleep efficiency, total sleep time, and duration of N3 significantly. This discrepancy between subjective and objective data might be attributed to the smaller sample size of PSG patients than PSQI patients as well as the minor differences in the areas of sleep improvement targeted by ALP and BC therapies.

With the improvement in insomnia, patients’ self-care ability has also been enhanced effectively. A previous study demonstrated that sleep disorders could impact the daytime functioning and quality of life (11), which was confirmed by improved daytime dysfunction of PSQI after treatment. Furthermore, the self-care ability can be enhanced by improving the nervous system and immune system and alleviating the basic diseases such as hypertension and diabetes (6, 7). Thus, the mechanism underlying the improvement of insomnia symptoms and self-care ability after stroke needs to be explored further along with the start time and course of treatment for PSI.

BC is a traditional Chinese patent medicine, composed of 15 Chinese herbal extractions. *Acanthopanax senticosus*, one of the main components of BC, plays an anxiolytic role *via* the regulation of autonomic function and increases the signal of the hippocampus *via* brain-derived neurotrophic factor (BDNF) (27). Semen Ziziphi spinosae (*Suanzaoren* in Chinese) and Radix et Rhizoma Salviae miltiorrhizae (*Danshen* in Chinese), the other two major components of BC, are well-known conventional herbal drugs in traditional Chinese medicine and have been used widely for the treatment of insomnia (28). The main compounds of Semen Ziziphi spinosae include saponins, swertisin, and fatty oils (29). The oral administration of spinosin and swertisin prolongs the sleeping time *via* a serotonergic mechanism (30). Moreover, the pharmacological activities of Radix et Rhizoma Salviae miltiorrhizae exert anti-inflammatory and cardioprotective effects, rendering it preferable for the treatment of insomnia coupled with inflammation or cardiovascular diseases (28). Polygalae Radix, also a main component of BC, is primarily used for treating insomnia and depression (31). Recent pharmacological studies in animals have demonstrated that the constituents of Polygalae Radix can improve cognition and potentially exert antipsychotic, antioxidant, and anti-inflammatory effects (31). Taken together, BC might improve the total sleep time of PSI through multiple targets and mechanisms.

In consideration of the mild and slow synergistic effects of the various herbal components, which are not conducive to inducing sleep directly, low-dose ALP plays a complementary but vital role and effectively reduces the latency of sleep. Also, only fewer side effects are observed in the low-dose ALP.

Nevertheless, the present study has several limitations: 1) The size of the sample is small, and the variability of the individual result is high. 2) It is a retrospective study, lacking long-term follow-up data for some patients. Thus, further randomized controlled trials are essential to clarify the efficiency and mechanism of BC on PSI. 3) A comprehensive evaluation of the dose and treatment duration of BC is absent in this study, and thus, future randomized controlled trials are needed to address these issues. 4) Although the evidence of using ALP in PSI is insufficient, it is still widely used in clinics due to its cost-efficiency. In recent years, with the improving economy, the use of eszopiclone has been increasing gradually. Thus, choosing eszopiclone as control and combination drug would seem to be a viable alternative.

## Ethics Statement

This study was approved by the ethics committee of The General Hospital of Western Theater Command, and received informed consent of patients and their families and signed informed consent.

## Author Contributions

QW designed the study and made critical revision of the article; JW and ZW collected data and drafted the article; XW analyzed and interpreted the data; GD, BZ, and YL collected data.

## Conflicts of Interest Statement

The authors declare that the research was conducted in the absence of any commercial or financial relationships that could be construed as a potential conflict of interest.

## References

[1] Mozaffarian D Benjamin EJ Go AS Arnett DK Blaha MJ Cushman M Heart disease and stroke statistics—2015 update: a report from the American Heart Association. Circulation (2015) 131(4):e29–322. 10.1161/CIR.0000000000000152 25520374

[2] Tsai CF Thomas B Sudlow CL Epidemiology of stroke and its subtypes in Chinese vs white populations: a systematic review. Neurology (2013) 81(3):264–72. 10.1212/WNL.0b013e31829bfde3 PMC3770160 23858408

[3] Centers for Disease Control and Prevention Prevalence and most common causes of disability among adults—United States, 2005. MMWR Morb Mortal Wkly Rep (2009) 58(16):421–6. 19407734

[4] Sterr A Kuhn M Nissen C Ettine D Funk S Feige B Post-stroke insomnia in community-dwelling patients with chronic motor stroke: physiological evidence and implications for stroke care. Sci Rep (2018) 8(1):8409. 10.1038/s41598-018-26630-y 29849087 PMC5976765

[5] Pasic Z Smajlovic D Dostovic Z Kojic B Selmanovic S Incidence and types of sleep disorders in patients with stroke. Medicinski arhiv (2011) 65(4):225–7. 10.5455/medarh.2011.65.225-227 21950229

[6] Dobkin BH Clinical practice. N Engl J Med (2005) 352(16):1677–84. 10.1056/NEJMcp043511 PMC4106469 15843670

[7] Scheff JD Calvano SE Lowry SF Androulakis IP Modeling the influence of circadian rhythms on the acute inflammatory response. J Theor Biol (2010) 264(3):1068–76. 10.1016/j.jtbi.2010.03.026 20307551

[8] Altemus M Rao B Dhabhar FS Ding W Granstein RD Stress-induced changes in skin barrier function in healthy women. J Investig Dermatol (2001) 117(2):309–17. 10.1046/j.1523-1747.2001.01373.x 11511309

[9] Kundermann B Krieg JC Schreiber W Lautenbacher S The effect of sleep deprivation on pain. Pain Res Manag (2004) 9(1):25–32. 10.1155/2004/949187 15007400

[10] Taylor DJ Lichstein KL Durrence HH Reidel BW Bush AJ Epidemiology of insomnia, depression, and anxiety. Sleep (2005) 28(11):1457–64. 10.1093/sleep/28.11.1457 16335332

[11] Kyle SD Morgan K Espie CA Insomnia and health-related quality of life. Sleep Med Rev (2010) 14(1):69–82. 10.1016/j.smrv.2009.07.004 19962922

[12] Cui JF Yang W Xie YM Sun Y Zhuang Y Wang YY Real-world analysis of concurrent diseases and medicine use among patients with insomnia. Zhongguo Zhong Yao Za Zhi (2014) 39(18):3519–26. 10.4268/cjcmm20141819 25532388

[13] Verster JC Volkerts ER Clinical pharmacology, clinical efficacy, and behavioral toxicity of alprazolam: a review of the literature. CNS Drug Rev (2004) 10(1):45–76. 10.1111/j.1527-3458.2004.tb00003.x 14978513 PMC6741717

[14] Khare A Thada B Jain N Singh D Singh M Sethi SK Comparison of effects of oral melatonin with oral alprazolam used as a premedicant in adult patients undergoing various surgical procedures under general anesthesia: a prospective randomized placebo-controlled study. Anesth Essays Res (2018) 12(3):657–62. 10.4103/aer.AER_90_18 PMC6157235 30283171

[15] Yang AL Liang QH Cui HJ Zhou HJ Luo JK Tang T Angiogenesis opens a way for Chinese medicine to treat stroke. Chin J Integr Med (2013) 19(11):815–9. 10.1007/s11655-013-1342-1 24170630

[16] Bu Y Lee K Jung HS Moon SK Therapeutic effects of traditional herbal medicine on cerebral ischemia: a perspective of vascular protection. Chin J Integr Med (2013) 19(11):804–14. 10.1007/s11655-013-1341-2 24170629

[17] Bian Y Tang X Mechanism of Bailemian capsules in the treatment of insomnia in mice. Zhonghua Yi Xue Za Zhi (2014) 94(46):3671–4. 10.3760/cma.j.issn.0376-2491.2014.46.015 25622963

[18] Capsules EGoCASoB Expert suggestions on clinical application of Bailemian capsules. J Neurosci Ment Health (2016) 16(2):142–4. 10.3969/j.issn.1009-6574.2016.02.006

[19] Bassetti CL Hermann DM Sleep and stroke. Handb Clin Neurol (2011) 99:1051–72. 10.1016/B978-0-444-52007-4.00021-7 21056242

[20] Huang QL Gao D Yue FG Jiang CG Zhang T Lei L Efficacy of Bailemian capsule combined with self-help cognitive behavioral therapy in treatment of chronic insomnia. Zhonghua Yi Xue Za Zhi (2016) 96(36):2893–7. 10.3760/cma.j.issn.0376-2491.2016.36.010 27760634

[21] Jauch EC Saver JL Adams HP Jr. Bruno A Connors JJ Demaerschalk BM Guidelines for the early management of patients with acute ischemic stroke: a guideline for healthcare professionals from the American Heart Association/American Stroke Association. Stroke (2013) 44(3):870–947. 10.1161/STR.0b013e318284056a 23370205

[22] Chen YK Lu JY Mok VC Ungvari GS Chu WC Wong KS Clinical and radiologic correlates of insomnia symptoms in ischemic stroke patients. Int J Geriatr Psychiatry (2011) 26(5):451–7. 10.1002/gps.2547 20845399

[23] Zhang S Chang C Zhang J Song B Fang H Xu Y Correlation analysis of sleep quality and youth ischemic stroke. Behav Neurol (2014) 2014:246841. 10.1155/2014/246841 25161340 PMC4139019

[24] Arzt M Young T Peppard PE Finn L Ryan CM Bayley M Dissociation of obstructive sleep apnea from hypersomnolence and obesity in patients with stroke. Stroke (2010) 41(3):e129–34. 10.1161/STROKEAHA.109.566463 PMC4230450 20075361

[25] Agnew HW Jr. Webb WB Williams RL The first night effect: an EEG study of sleep. Psychophysiology (1966) 2(3):263–6. 10.1111/j.1469-8986.1966.tb02650.x 5903579

[26] Pinto LR Jr. Silva AB Tufik S Rapid eye movements during paradoxical sleep in patients with cerebrovascular disease. Arq Neuropsiquiatr (2000) 58(2A):239–45. 10.1590/S0004-282X2000000200006 10849621

[27] Miyazaki S Oikawa H Takekoshi H Hoshizaki M Ogata M Fujikawa T Anxiolytic effects of Acanthopanax senticosus harms occur via regulation of autonomic function and activate hippocampal BDNF(-)TrkB signaling. Molecules (2018) 24(1):132. 10.3390/molecules24010132 PMC6337493 30602695

[28] Fang X Hao JF Zhou HY Zhu LX Wang JH Song FQ Pharmacological studies on the sedative-hypnotic effect of Semen Ziziphi spinosae (Suanzaoren) and Radix et Rhizoma Salviae miltiorrhizae (Danshen) extracts and the synergistic effect of their combinations. Phytomedicine (2010) 17(1):75–80. 10.1016/j.phymed.2009.07.004 19682877

[29] Zhang M Zhang Y Xie J Simultaneous determination of jujuboside A, B and betulinic acid in semen Ziziphi spinosae by high performance liquid chromatography–evaporative light scattering detection. J Pharm Biomed Anal (2008) 48(5):1467–70. 10.1016/j.jpba.2008.09.022 18977107

[30] Wang LE Bai YJ Shi XR Cui XY Cui SY Zhang F Spinosin, a C-glycoside flavonoid from semen Zizhiphi Spinozae, potentiated pentobarbital-induced sleep via the serotonergic system. Pharmacol Biochem Behav (2008) 90(3):399–403. 10.1016/j.pbb.2008.03.022 18466960

[31] Ling Y Li Z Chen M Sun Z Fan M Huang C Analysis and detection of the chemical constituents of Radix Polygalae and their metabolites in rats after oral administration by ultra high-performance liquid chromatography coupled with electrospray ionization quadrupole time-of-flight tandem mass spectrometry.J Pharm Biomed Anal (2013) 85:1–13. 10.1016/j.jpba.2013.06.011 23860503

